# Temperature-to-Digital Converters’ Evolution, Trends and Techniques across the Last Two Decades: A Review

**DOI:** 10.3390/mi13112025

**Published:** 2022-11-19

**Authors:** Antonio Aprile, Edoardo Bonizzoni, Piero Malcovati

**Affiliations:** Department of Electrical, Computer and Biomedical Engineering, University of Pavia, 27100 Pavia, Italy

**Keywords:** temperature, TDCs, smart sensors, design trends

## Abstract

This paper presents an extensive review of the main highlights in the Temperature-to-Digital Converters (TDCs) field, which has gained importance and research interest throughout the last two decades. The key techniques and approaches that have led to the evolution of this kind of systems are presented and compared; their peculiarities are identified in order to highlight the pros and cons of the different design methods, and the main trade-offs are extracted from this analysis. Finally, the trends that have emerged from the performance evaluation of the large amount of published works in this field are identified with the purpose of providing a directional view of the past, present and future features of these devices.

## 1. Introduction

On-chip temperature measurements have acquired an increasingly important role over the past two decades, especially if we consider sensors that produce data in the digital domain, referred to as Temperature-to-Digital Converters (TDCs). The growing computational power of modern microprocessors has given rise to a higher degree of criticality in their thermal management process [1]; for instance, dynamic voltage and frequency scaling (DVFS), a commonly used approach in this framework [2], requires responsive temperature tracking to allow an effective control on the thermal status of the microprocessor and, furthermore, the cooling fans’ speed regulation is also based on a continuous temperature monitoring [3,4,5]. Another field that has featured a remarkable growth in recent years is the Micro-Electro-Mechanical Systems (MEMS) one [6]; the employment of these devices for Internet of Things (IoT) applications, supported by a parallel technological development, has led the research focus to more and more robust devices with respect to the influence of environmental effects. One of the main challenges is, indeed, to mitigate the impact of the ambient temperature on the performance of these devices; the micro-structures used as sensing elements suffer from a significant thermal spread causing a degradation of the reliability of the sensed quantity. For this reason, high-precision MEMS devices also require a temperature tracking to compensate for the drift of their parameters [7,8,9,10,11]. Integrated temperature sensors are also used for clinical applications [12,13,14]; devices that provide a high accuracy monitoring in the human body temperature range are needed for the detection of atypical biomedical conditions. Lastly, since temperature is a fundamental physical parameter of both industry and everyday life, on-chip temperature measurements are also combined with radio-frequency identification (RFID) tags in several applications: monitoring of the food cold chain [15,16], environmental monitoring [17,18], supply chain management of healthcare products [19], animal healthcare monitoring [20] and many more.

This paper, besides proposing a State-of-the-Art analysis, reviews the different design techniques employed for all the presented on-chip temperature sensing applications and is organized as follows: Section 2 addresses the basics of TDCs taking all their relevant parameters into account and explaining, through four different subsections, the different design techniques adopted so far. Section 3, instead, is focused on the main trends and trade-offs that emerge from the analysis of the previous section; its goal is to provide an overview of the TDC features’ evolution over more than twenty years of research activity and to deliver to the reader an useful set of performance considerations to discerningly start a new design in this framework or simply to enter more deeply into the world of TDCs. Section 4 concludes the paper, highlighting the main introduced concepts with a brief recap.

## 2. Temperature-to-Digital Converters: Theory and Design Techniques

**Figure 1:**
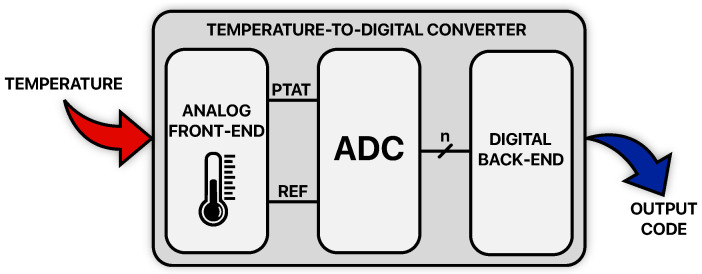
Conceptual diagram of a Temperature-to-Digital Converter.

There are a lot of applications requiring on-chip temperature sensing, as seen in the introduction, and concern several systems in the microelectronics field; despite their wide range, all the reported examples [3,4,5,7,8,9,10,11,12,13,14,15,16,17,18,19,20] have one important feature in common: they provide temperature information in the form of digital data. This is fundamental as it makes them compatible for a direct communication with digital signal processing (DSP) circuits that can easily handle the needed temperature information and at the same time reduces the complexity of the system they are inserted in; for this reason, they are often referred to as *smart temperature sensors* [21] or as *Temperature-to-Digital Converters (TDCs)*. It is important to specify that this category of temperature sensors was born with a cost-minimization perspective and that its development in the past two decades has consequently followed this line; even if, in principle, these fully integrated temperature sensors have significant limitations in terms of accuracy and sensing range with respect to other existing discrete sensors, their great success is related to their compatibility with large-scale production of low-cost products being integrated within the system in which they are operated. Figure 1 shows the conceptual diagram of a TDC.

It is composed of an Analog Front-End (AFE), an Analog-to-Digital Converter (ADC) and a Digital Back-End (DBE). The TDC’s input signal is temperature; the AFE, the first block of the chain, is responsible to sense it achieving an electrical form for it (either in the voltage or in the current domain) and to generate at its output the signals needed for the Analog-to-Digital conversion: a proportional-to-absolute-temperature (PTAT) signal which contains the information to be converted and a reference (REF) signal, which in principle is a Zero-Temperature-Coefficient (ZTC) signal, with respect to which the conversion is carried out. Those signals enter the ADC which produces PTAT digital words with an intrinsic *n*-bit resolution and with a data rate ($f_{S}$) that depends on the converter architecture; this operation is typically performed without the use of sample and hold (S/H) circuits because of the relative slowness of the temperature signal with respect to the common conversion rates of ADCs. The *n*-bit codes are then processed by the DBE that, in fact, acts as an oversampler; it refines their intrinsic resolution performing decimation and filtering with a certain OverSampling Ratio (OSR) in order to obtain the output codes of the TDC which feature a higher resolution at the cost of a lower data rate ($f_{S}/OSR$).

The resulting time interval required to perform a single Temperature-to-Digital conversion is therefore given by

(1) $$T_{conv}=\frac{1}{f_{S}\cdot OSR}.$$

Considering the TDC’s minimum working supply voltage ($V_{sy}$) and the current drained from it ($I_{sy}$), its conversion energy can be defined as

(2) $$E_{conv}=V_{sy}\cdot I_{sy}\cdot T_{conv}.$$

It is a parameter of paramount importance together with the TDC’s resolution (Res) which is the minimum temperature difference that can correctly be detected and which is determined by the quantization noise of the ADC, by the electronic noise (thermal, flicker, etc.) and by $T_{conv}$ itself. Another parameter of interest is the temperature inaccuracy (IA); in absolute form, it is a statistical evaluation of the worst case (or ±3σ) temperature error and, introducing the TDC conversion range ($T_{range}$), its relative form can be expressed as

(3) $$IA_{rel}=\frac{IA}{T_{range}}.$$

This quantity is strongly dependent on the number of controlled temperatures at which the TDC gets trimmed ($n_{trim}$) [22,23], an unavoidable procedure in most applications; the trimming process, which basically consists of calibrating the sensed temperature error, is a cost of great relevance in the TDC framework as heating and cooling the devices to be trimmed is a very time consuming operation. For this reason, $n_{trim}$ should be minimized to preserve the cost-effectiveness of the sensor.

Due to the presence of this great variety of parameters of interest, several Figures of Merit (FoMs) have been introduced to provide TDC performance metrics in a synthetic way and from specific perspectives:

(4) $$FoM_{Res}=E_{conv}\cdot {\left(Res\right)}^{2},$$

(5) $$FoM_{IA}=E_{conv}\cdot {\left(IA_{rel}\right)}^{2},$$

(6) $$FoM_{\$}=\left(1+n_{trim}\right)\cdot \sqrt{\frac{Area}{F^{2}}},$$

(7) $$FoM_{global}=\frac{E_{conv}\cdot Res\cdot IA}{{T_{range}}^{2}}\cdot \left(1+n_{trim}\right)\cdot \sqrt{\frac{Area}{F^{2}}}.$$

(4) and (5), presented in [24], involve the TDC conversion energy together with its resolution or its inaccuracy, respectively. (6), instead, addresses only the production cost of the TDC (Area is the active silicon area of the device, *F* is the feature size of the adopted technological process) while (7) provides a global overview of the TDC performance [25].

Several ADC architectures have been used, in literature, to be included in TDCs; there are examples of Flash-based TDCs [26,27], of SAR-based ones [11,28], of ΣΔ-based ones [5,14], of time/frequency-domain-based ones [22,29] or of hybrid solutions [30,31]. It is important to notice that even if, conceptually, Flash ADCs and SAR ADCs are faster for a given quantization noise and clock frequency, to overcome the limits imposed by the presence of thermal noise, their output codes still need to be processed by the DBE and therefore, for the same amount of power consumption, are not automatically at a higher energy efficiency level with respect to the ΣΔ-based or the time/frequency-domain-based alternatives. Actually, thanks to their versatility, ΣΔ converters are the most used ones in the case of AFEs generating static temperature-dependent signals while time/frequency-domain-based ADCs are preferred in the case of dynamic temperature-dependent signals.

It makes sense to categorize TDCs on the basis of the sensing device/technique adopted within the AFE; four main categories can be identified: BJT-based TDCs (Section 2.1), MOS-based TDCs (Section 2.2), resistor-based TDCs (Section 2.3) and Thermal Diffusivity (TD) based TDCs (Section 2.4). The next subsections address in detail the peculiarities of each of these sensing techniques.

### 2.1. BJT-Based TDCs

**Figure 2:**
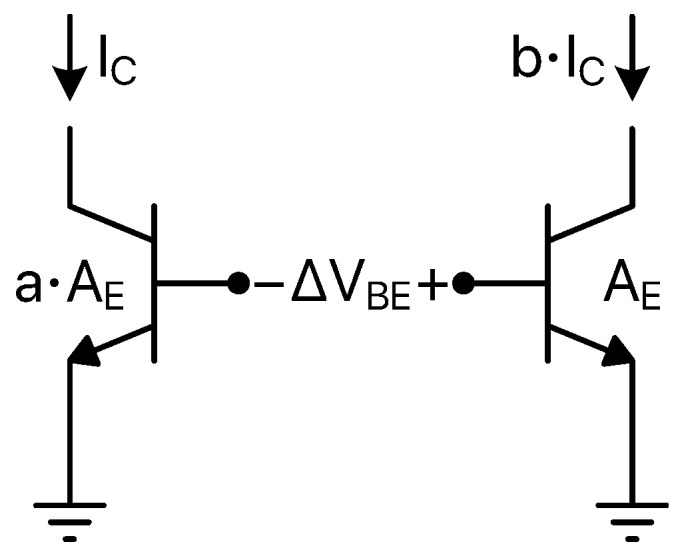
BJT pair for $\Delta V_{BE}$ signal generation.

On-chip temperature sensing can be achieved exploiting the thermal behaviour of the base-to-emitter voltage ($V_{BE}$) of bipolar transistors operated in the forward-active region [4,5,11,13,16,19,26,32,33,34,35,36,37,38,39,40,41,42,43,44,45,46,47]. It can be expressed as

(8) $$V_{BE}=\frac{kT}{q}ln\left(\frac{I_{C}}{I_{S}}\right)$$

where *k* is the Boltzmann constant, *T* is the absolute temperature, *q* is the magnitude of the elementary charge, $I_{C}$ is the collector current and $I_{S}$ is the bipolar saturation current which, typically, is in the fA to pA range, is proportional to the emitter area and exhibits a strong temperature dependence (as a rule of thumb, it doubles for every 5 K rise). This provides a complementary-to-absolute-temperature (CTAT) voltage variation with the well-known average slope of about −2 mV/K. Considering a pair of BJTs operating at different collector currents and/or having different emitter areas, a proportional-to-absolute-temperature (PTAT) signal is obtained taking the difference of their base-to-emitter voltages into account. According to the scheme and the notations of Figure 2, the following expression holds:

(9) $$\Delta V_{BE}=\frac{kT}{q}ln\left(a\cdot b\right)$$

where *a* and *b* are the emitter areas and collector currents ratios, respectively.

Referring to Figure 1, a $\Delta V_{BE}$-dependent signal can be used as the PTAT one while the REF signal can be generated by means of a proper combination of $V_{BE}$-dependent and $\Delta V_{BE}$-dependent contributions [48].

BJT-based TDCs are the most common ones thanks to the good intrinsic accuracy of bipolar transistors [49]; this leads to temperature sensors requiring at most one trimming point to achieve inaccuracy values which other sensing techniques implement after two trimming points or more. This feature is essential from the cost-effectiveness point of view and, together with the availability of bipolar transistors (even if parasitic) within most CMOS processes, is the reason for the great employment of these kinds of devices for on-chip temperature sensing.

### 2.2. MOS-Based TDCs

Another possibility for integrated temperature sensing is to rely on the thermal variations related to MOS devices; an option is to exploit the significant temperature dependence offered by the gate-to-source voltage ($V_{GS}$) of transistors operated in the subthreshold region [29,50,51,52,53]:

(10) $$V_{GS}=V_{th}+\frac{nkT}{q}ln\left(\frac{I_{D}}{I_{D0}}\right)$$

where $V_{th}$ is the threshold voltage, *n* depends on the MOS structure and $I_{D0}$ is the drain current for $V_{GS}$ = $V_{th}$. Besides being directly proportional to the transistor aspect ratio (W/L), $I_{D0}$ increases with temperature almost parabolically giving rise to a CTAT behaviour for $V_{GS}$; in absolute value, it exhibits a slightly lower average slope (about −1.5 mV/K [49]) with respect to the previously introduced $V_{BE}$ slope (about −2 mV/K). Similarly to the BJT case, considering a pair of MOSFETs biased at different drain currents and/or having different aspect ratios, a PTAT signal is obtained taking the difference of their gate-to-source voltages into account.

**Figure 3:**
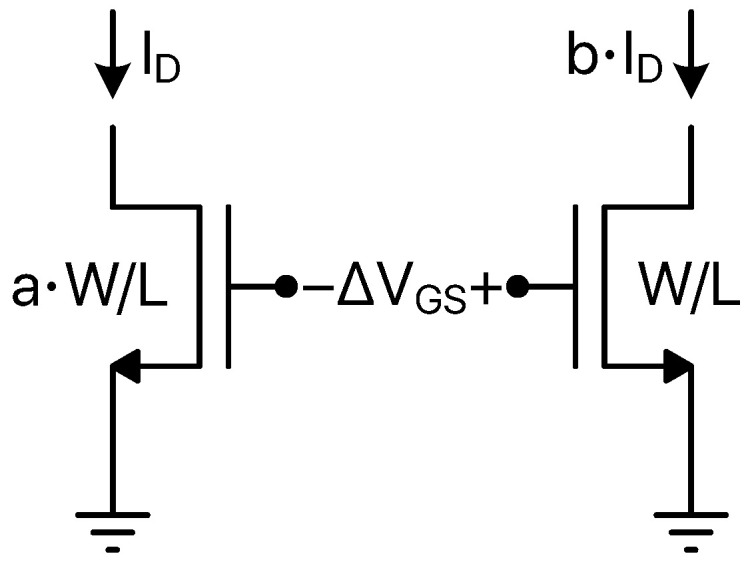
MOSFET pair for $\Delta V_{GS}$ signal generation.

According to the scheme and the notations of Figure 3, the $\Delta V_{GS}$ signal can be expressed as

(11) $$\Delta V_{GS}=\frac{nkT}{q}ln\left(a\cdot b\right)$$

where *a* and *b* are the W/L and drain currents ratios, respectively. It is interesting to notice that the PTAT sensitivity offered by subthreshold operated MOS devices benefits from the presence of the *n* coefficient if compared to the bipolar case; considering that this technology dependent parameter is intrinsically larger than 1, for the same *a* and *b* ratios, the $\Delta V_{GS}$ temperature sensitivity is intrinsically higher than the $\Delta V_{BE}$ one [49]. Also in this case, a reference signal can be generated by combining $V_{GS}$-dependent and $\Delta V_{GS}$-dependent contributions.

Another option to exploit the temperature dependence of MOS devices for on-chip sensing is to consider the propagation time ($t_{p}$) of CMOS inverters; as shown in (12), this parameter depends on many variables such as the adopted supply voltage ($V_{DD}$), the threshold voltage ($V_{th}$) and the size (*W*,*L*) of the devices constituting the inverter, the carriers mobility (μ), the oxide capacitance ($C_{ox}$) and the capacitance ($C_{L}$) of the load to be driven:

(12) $$t_{p}=f\left(V_{DD},V_{th}\left(T\right),\mu \left(T\right),W,L,C_{ox},C_{L}\right).$$

In particular, $V_{th}$ and μ are a function of the temperature that, if properly exploited, may lead to an effective sensing.

**Figure 4:**
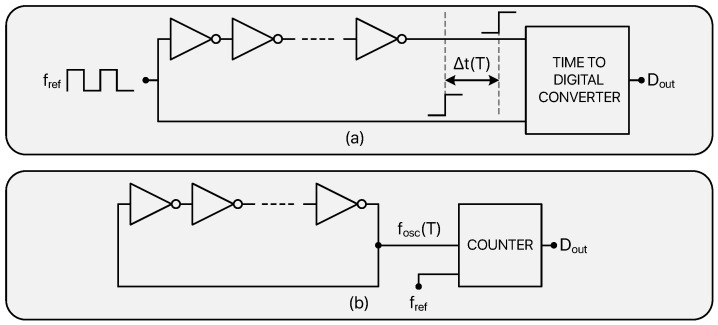
Main circuits that exploit the temperature dependency of the propagation time of CMOS inverters to achieve a Temperature-to-Digital conversion: delay line based TDC (**a**), ring oscillator based TDC (**b**).

The first way to achieve a $t_{p}$-based temperature to digital conversion is to rely on a delay line [29,30,54,55,56,57] as shown in Figure 4a. A clock signal running at a reference frequency ($f_{ref}$) is passed through a delay line composed by *N* inverters and is compared with an undelayed version of itself; this gives rise to temperature dependent time intervals which can be expressed as

(13) $$\Delta t\left(T\right)=N\cdot t_{p}\left(T\right),$$

and which are processed by a time-to-digital converter that, hence, generates temperature dependent digital words ($D_{out}$).

The second possibility, instead, is to exploit the thermal behaviour of ring oscillators [22,52,53,58,59,60,61,62,63,64] as shown in Figure 4b in which the $t_{p}$ temperature dependence impacts the oscillation frequency ($f_{osc}$) as shown by the following expression:

(14) $$f_{osc}\left(T\right)=\frac{1}{2t_{p}\left(T\right)N}.$$

The signal produced by the oscillator gets processed by a counter (clocked at $f_{ref}$) which generates temperature dependent digital codes ($D_{out}$) depending on the oscillations count. In addition to this, in 2019, new interesting MOS-based techniques were proposed, opening the doors for sub-nW TDCs design. An innovative temperature sensing principle based on the gate-leakage current of MOS devices was adopted in [65,66], resulting in an exceptionally low power consumption. The $t_{p}$-based and the leakage-based approaches offer outstanding performance in terms of energy/conversion but typically exhibit poor linearity and accuracy.

### 2.3. Resistor-Based TDCs

Also integrated resistors exhibit a significant thermal variability that makes them suitable for on-chip temperature sensing. Considering a first order approximation, their resistance value can be expressed as

(15) $$R=R_{0}\left(1+TC\cdot \Delta T\right),$$

where $R_{0}$ is the resistance value at a reference temperature $T_{0}$, TC is the temperature coefficient and

(16) $$\Delta T=T-T_{0}.$$

Table 1 and Table 2 report realistic TC values for some kinds of resistors in 0.18-µm and 65-nm CMOS processes, respectively.

**Figure 5:**
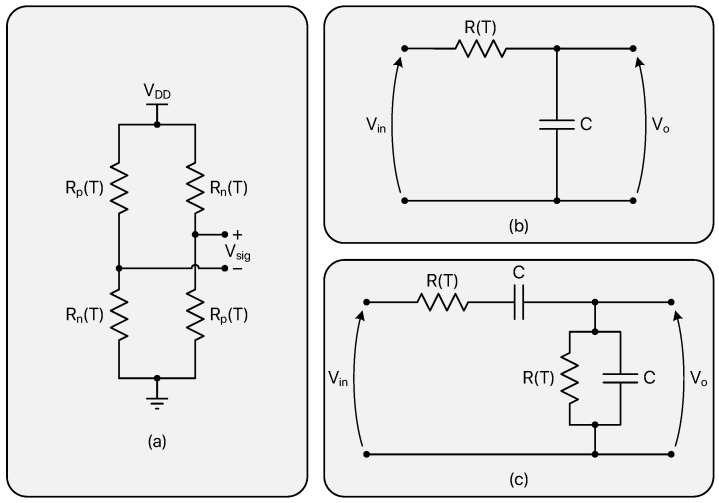
Main circuits used to extract temperature information from the thermal behaviour of integrated resistors: Wheatstone bridge (**a**); RC filter (**b**); Wien-bridge filter (**c**).

In the last decade, three main techniques have been exploited to electronically benefit from the temperature dependence of such resistors: Wheatstone bridges, RC filters and Wien-bridge filters. Figure 5 illustrates the basic schemes of these sensing possibilities.

TDCs based on Wheatstone bridges [28,31,69,70,71,72] typically rely on the combined effect of a positive TC resistor ($R_{p}$) and of a negative TC one ($R_{n}$).

(17) $$R_{p}=R_{0}\left(1+\alpha \Delta T\right),\;\alpha >0,$$

(18) $$R_{n}=R_{0}\left(1+\beta \Delta T\right),\;\beta <0.$$

According to Figure 5a, temperature information is contained in the $V_{sig}$ voltage, which can be expressed as

(19) $$V_{sig}=\frac{R_{p}-R_{n}}{R_{p}+R_{n}}\cdot V_{DD}=\frac{R_{0}\left(1+\alpha \Delta T\right)-R_{0}\left(1+\beta \Delta T\right)}{R_{0}\left(1+\alpha \Delta T\right)+R_{0}\left(1+\beta \Delta T\right)}\cdot V_{DD}=\frac{(\alpha -\beta )\Delta T}{2+(\alpha +\beta )\Delta T}\cdot V_{DD}.$$

**Figure 6:**
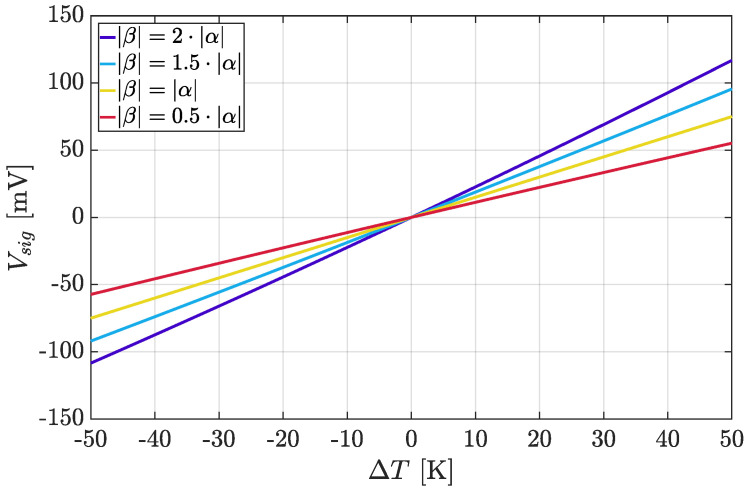
$V_{sig}$ as a function of ΔT in different (|α|;|β|) conditions for $\alpha =+1.5\cdot 10^{-3}$ K${}^{-1}$, realistic value for n+ diffusion integrated resistors [67].

Figure 6 shows $V_{sig}$ as a function of temperature for several (|α|;|β|) combinations in a symmetrical 100 K ΔT range; to maximize the Wheatstone bridge temperature sensitivity, the |β|/|α| ratio should be selected as high as possible according to the resistor availability of the adopted technology. On the other hand, as pointed out by Table 3 and as can be easily derived from (19), the linearity of the thermal response degrades moving away from the |α|=|β| optimal case (it is important to mention that the reported considerations do not take any second or higher order contribution to the resistance temperature variability into account).

TDCs based on RC [68,73,74] and Wien-bridge [67,75,76,77,78] filters, instead, take advantage of the temperature variations of their transfer functions; in both cases, as can be deduced from Figure 5b,c, the temperature dependence of the employed resistors causes an alteration of their phase response that can be exploited to achieve the desired temperature-to-digital conversion; this is achieved by driving the considered structures with signals oscillating close to the fundamental frequency of the filters ($\omega_{0}=1/R\left(T\right)C$ in both cases) at room temperature and processing their output by means of appropriate phase-to-digital conversion circuits. Given the RC transfer function,

(20) $$H\left(j\omega \right)=\frac{1}{1+j\omega R(T)C},$$

its temperature-dependent phase shift can be expressed as

(21) $$\phi \left(j\omega \right)=-\arctan\left(\omega R(T)C\right).$$

**Figure 7:**
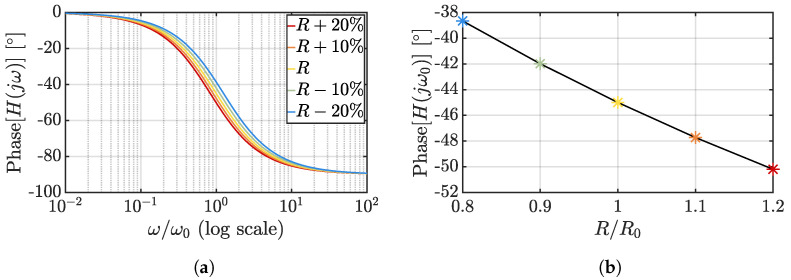
Phase dependency on resistance variations for an RC filter: impact on the phase response of the filter (**a**) and phase shift at $\omega_{0}$ as a function of the resistance variation (**b**).

Figure 7a shows the RC phase response for different resistance values in the ±20% range where the selected colors conceptually refer to a positive TC resistor (the warmer the color, the higher the temperature); since the most effective temperature phase impact occurs at $\omega =\omega_{0}$, Figure 7b reports the phase shift generated by the RC filter as a function of the resistance variation with respect to the room temperature value ($R_{0}$).

The Wien-bridge transfer function is instead given by

(22) $$H_{WB}\left(j\omega \right)=\frac{j\omega R(T)C}{1-\omega^{2}R^{2}\left(T\right)C^{2}+3j\omega R\left(T\right)C},$$

and its temperature-dependent phase shift can be expressed as

(23) $$\phi_{WB}\left(j\omega \right)=-\arctan\left(\frac{\omega^{2}R^{2}\left(T\right)C^{2}-1}{3\omega R(T)C}\right).$$

**Figure 8:**
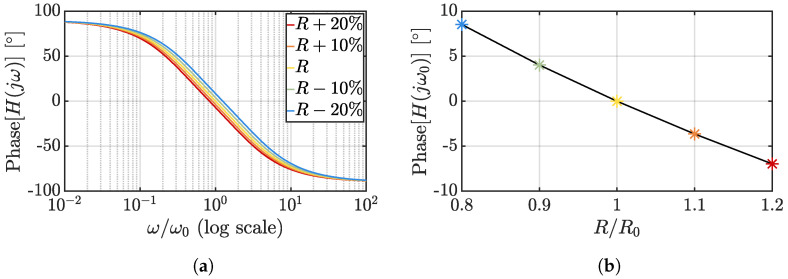
Phase dependency on resistance variations for a Wien-bridge filter: impact on the phase response of the filter (**a**) and phase shift at $\omega_{0}$ as a function of the resistance variation (**b**).

In keeping with the graphs reported for the RC case, Figure 8a shows the Wien-bridge phase response for the same resistance value variation range while Figure 8b reports the resulting phase shift at $\omega_{0}$.

For the same resistance variation and capacitor value (*C*), the Wien-bridge filter achieves a better phase sensitivity to temperature and linearity if compared to the RC one at the cost of a double occupied area; considering that, typically, the size of the filter is not the limiting element in the TDC area breakdown, Wien-bridge filters are the preferred choice over RC ones.

As will be addressed in Section 3.4, the TDCs exploiting the presented resistor-based temperature sensing techniques are undoubtedly the best in class from the energy efficiency point of view but typically are less accurate than BJT-based solutions and more power hungry than MOS-based solutions.

### 2.4. TD-Based TDCs

**Figure 9:**
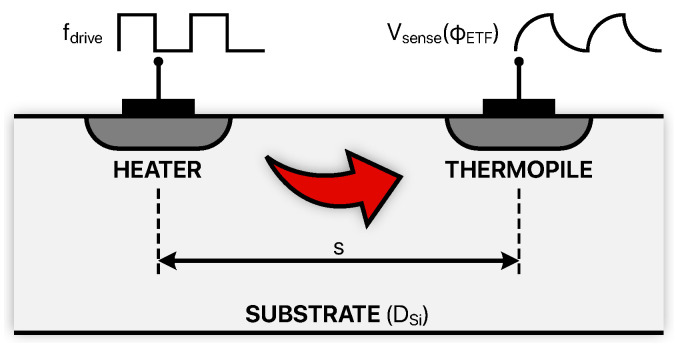
Electrothermal filter for thermal diffusivity measurement.

The last considered category is that of thermal diffusivity TDCs [79,80,81,82,83,84]. These on-chip sensors exploit measurements of the thermal diffusivity of silicon ($D_{Si}$) which exhibits a considerable temperature dependence and, moreover, does not suffer from process spread variations. This quantity can be sensed by means of the electrothermal filter (ETF) shown in Figure 9.

A heater that can be realized by a diffusion resistor is driven by a square wave (at a $f_{drive}$ frequency) and, consequently, generates heat pulses which diffuse to a neighboring thermopile placed at a distance *s*; these pulses are affected by a delay and by an attenuation which are determined by $D_{Si}$ which, in turn, is a function of the temperature ($\propto T^{1.8}$) [81]. For this reason, the phase of the voltage sensed by the thermopile ($V_{sense}$) is sensitive to temperature and, according to [84], can be expressed as

(24) $$\phi_{ETF}\left(T\right)=-s\cdot \sqrt{\frac{f_{drive}}{2D_{Si}\left(T\right)}}.$$

With similar phase-to-digital conversion solutions as the ones needed for the previously introduced RC-based and Wien-bridge-based TDCs, $\phi_{ETF}$ can be digitized, thus generating temperature dependent digital codes. The major drawback of this kind of sensing technique is the large amount of power (>1 mW) burnt to drive the heater: its energy inefficiency makes it unsuitable for the majority of battery-powered applications. Nevertheless, TD-based TDCs offer a really remarkable accuracy performance, especially considering that, in many cases, no trimming procedure is required; this aspect will be further explored in Section 3.2.

## 3. State-of-the-Art Review and Design Trends

Over the past two decades, more than 150 TDC works have been published, each of which can be assigned to one of the four categories introduced in Section 2. A really valuable survey [85] that keeps track of all these works has been made available by prof. Makinwa from TU Delft and has been adopted as dataset for all the following analysis and considerations. The time evolution and the performance peculiarities of the four considered TDC types are investigated in the next subsections, each addressing a primary parameter of interest of TDCs: resolution (Section 3.1), inaccuracy (Section 3.2), conversion energy (Section 3.3), energy efficiency (Section 3.4) and silicon area (Section 3.5). All the reported trend-lines have been produced by a log-scale adapted smoothing spline method based on the geometric mean of the considered parameter values for each year.

### 3.1. Resolution

As introduced in Section 2, the resolution of a TDC is the minimum temperature difference that can correctly be detected; it is a function of the intrinsic quantization noise of the ADC used to perform the temperature-to-digital conversion, of the amount of electronic noise that affects the TDC output and of the DBE processing type.

**Figure 10:**
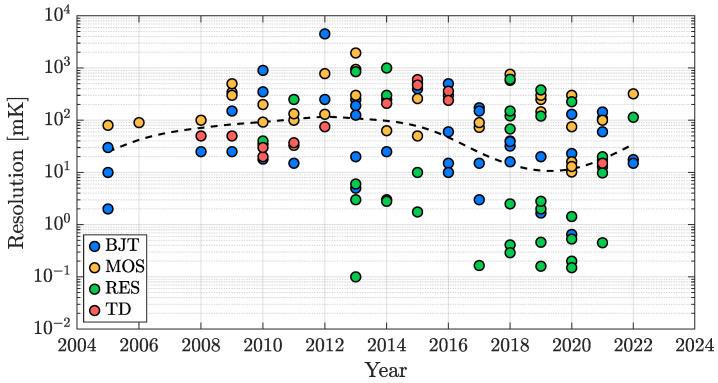
TDC resolution time evolution in the last two decades.

**Figure 11:**
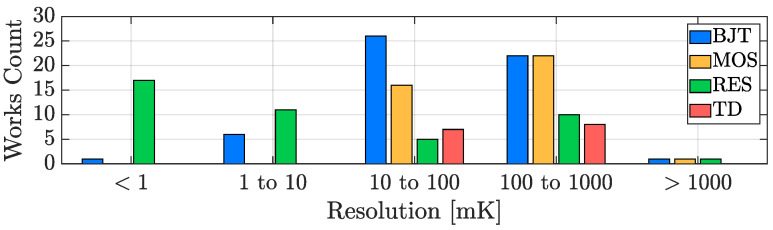
TDC resolution performance distribution with sensing-type distinction.

Figure 10 reports the resolution of the considered works as a function of the publication year for all of the four studied categories of sensors; it can be noticed that the resolution performance of TDCs is basically trend-less since its requirements are strongly application-dependent: the resolution specification is of prime importance in the cases in which the sensing goal is to precisely detect temperature variations but a moderate value can be acceptable in the case of accuracy-oriented designs, in favour of a conversion energy saving. In addition to this, it can be observed that the first examples of resistor-based TDCs have been introduced just starting from 2010 and, a few years later, a series of high resolution works exploiting this sensing approach has been proposed, actually showing their greater potential regarding the resolution parameter. This feature can be further appreciated, considering Figure 11; the resolution of each item shown in Figure 10 has been collected to build a bar plot organized on the basis of five decades: maintaining the sensing-type distinction, it provides an overview of how the resolution performance of all the considered works is distributed, confirming the advantage of resistor-based TDCs. It should be taken into account that, in principle, resolution can always be improved by increasing the DBE OSR at the cost of a higher conversion time (1) and that, therefore, the performance limitation of the other kinds of sensing approaches is actually related to their worse energy efficiency, a parameter that will be addressed in detail in Section 3.4.

### 3.2. Inaccuracy

**Figure 12:**
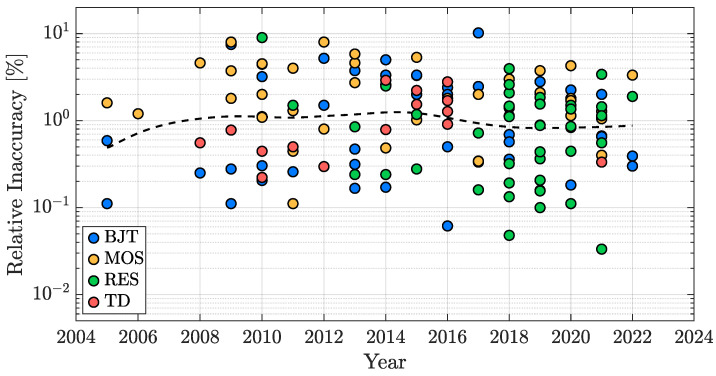
TDC inaccuracy time evolution in the last two decades.

In the same vein of what was presented for resolution, Figure 12 shows the relative inaccuracy, defined in (3), as a function of the publication year for the TDCs surveyed in [85]. Also in this case, a trend-less behaviour can be noticed, once again because of the application-dependency of the accuracy specification of TDCs. For example, the ones designed for clinical applications require absolute inaccuracy values on the order of ±0.1 °C, while the ones used to track the temperature status of microprocessors or to compensate for the thermal drift in MEMS resonators typically require an inaccuracy of about ±1 °C or even worse.

**Figure 13:**
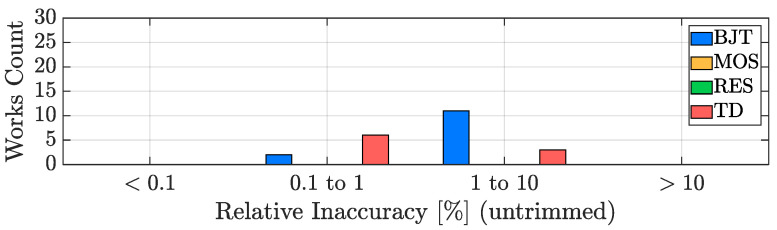
Untrimmed TDC inaccuracy performance distribution with sensing-type distinction.

**Figure 14:**
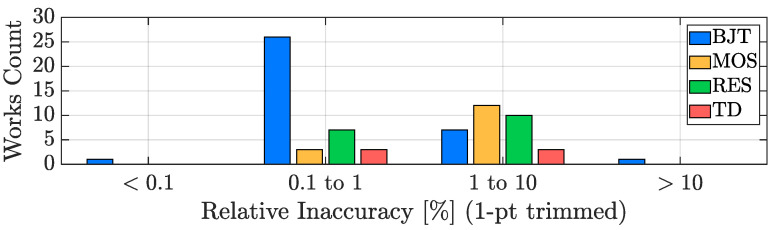
1-pt trimmed TDC inaccuracy performance distribution with sensing-type distinction.

**Figure 15:**
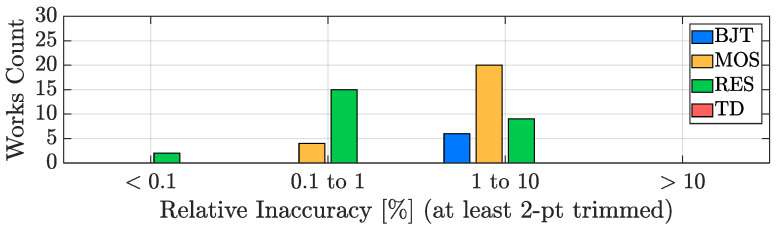
2-pt (or more) trimmed TDC inaccuracy performance distribution with sensing-type distinction.

In order to evaluate the accuracy performance potential of the four considered sensing techniques, it is of paramount importance to take the number of trimming points into account since, as a rule of thumb, the transition to the 1-point trimming condition from the untrimmed one typically provides a benefit of at least a factor two to the accuracy of the sensor, while the addition of a trimming point at a second temperature generally improves the TDC accuracy of at least an extra factor four. For this reason, the inaccuracy bar plot, analogous to the resolution one of Figure 11, has been split into three plots: Figure 13 addresses the untrimmed works, Figure 14 focuses on the TDCs with a single-temperature trimming, while Figure 15 considers the works with at least two trimming points.

It can be seen that, from the accuracy point of view, the TD-based TDCs are the best in class, followed by the BJT-based ones; they are, indeed, the only types of sensors that can achieve relatively good accuracy without the need of being trimmed (Figure 13), a huge advantage in terms of cost-effectiveness. MOS-based TDCs and resistor-based TDCs, instead, require at least one trimming point (in most cases 2-pts, Figure 15) to offer acceptable performance and therefore are undesirable for accuracy-oriented designs. On top of this, it is important to remember that, in addition to the spread due to the sensing element, inaccuracy is also determined by the spread of all the components present in the device [49] and consequently it may not be limited by the sensing technique choice but by the matching performance of the entire circuitry of the AFE and of the ADC. In this framework, a key element to take into account is the silicon area size of the TDC (addressed in Section 3.5): the smaller its active area, the tougher the achievement of acceptable accuracy values.

### 3.3. Conversion Energy

**Figure 16:**
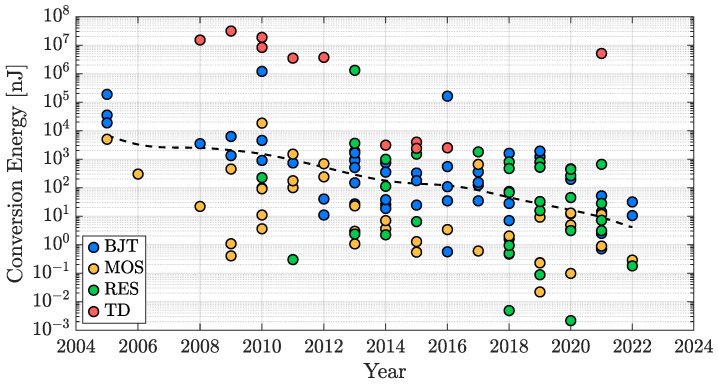
TDC conversion energy time evolution in the last two decades.

The growth of the IoT market and the increasing number of battery-powered systems requiring on-chip temperature sensing have induced a really strong trend when it comes to TDC conversion energy (2). This parameter, which is a full-fledged measure of the energy price to pay to achieve a single temperature-to-digital conversion, is crucial to ensure the highest battery lifetime possible or even to allow the operation of energy-harvesting-based devices such as [86], in which temperature-dependent digital codes are generated with just a few picojoules of energy. Figure 16 reports the conversion energy values of the same works analyzed in the previous subsections as a function of their publication year. In this case, a trend towards lower values is definitely visible; the TDC conversion energy exhibits a reduction of about a factor 10 every five years, a clear direction that allows for predicting the future evolution of these kinds of devices.

**Figure 17:**
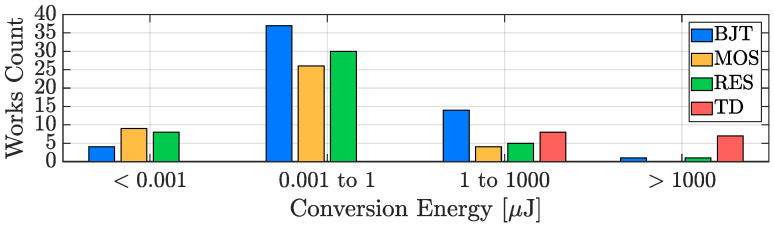
TDC conversion energy performance distribution with sensing-type distinction.

As reported for the resolution and the inaccuracy cases, Figure 17 shows the conversion energy performance distribution across four orders of magnitude and with the different sensing-types taken into account. It can be noticed that, undoubtedly, TD-based TDCs, due to the power consumed by the heater, require the highest conversion energy while the other three types exhibit quite similar performance. Similarly to the resolution discussion (Section 3.1), it is important to consider that, naturally, the conversion energy can be reduced by accepting a poorer temperature resolution and therefore, also in this case, the reported conversion energy values are linked to the efficiency of the different sensing techniques that will be addressed in the next subsection.

### 3.4. Energy Efficiency

**Figure 18:**
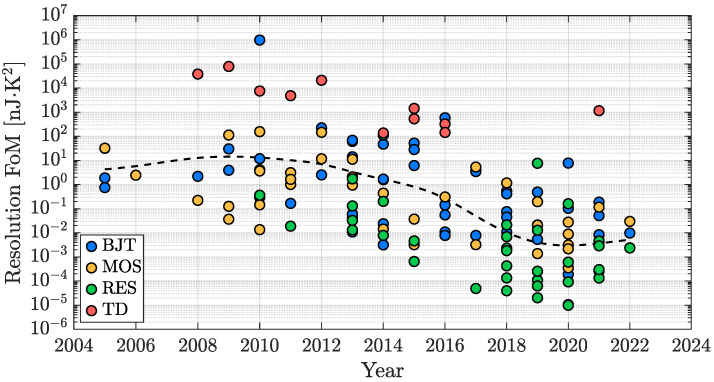
TDC energy efficiency time evolution in the last two decades.

Both Section 3.1 and Section 3.3 have introduced the resolution vs. conversion energy trade-off. The energy efficiency of a TDC is a metric of what resolution can be achieved for a given conversion energy or, on the other hand, what conversion energy is needed to achieve a target resolution. To determine what the trade space of a certain TDC is and, consequently, to determine its energy efficiency, it is useful to consider the resolution FoM introduced in (4), in which Res is squared because it is usually limited by thermal noise and therefore, to achieve an improvement of a factor two of it, a four times larger conversion time is required and so on. Figure 18 shows the time evolution of the energy efficiency of the same considered works of the previous subsections. Three different phases can be identified: at first, approximately until 2010, there is a horizontal phase in which the novelty of such kind of integrated sensors has resulted in TDCs without the primary target of energy efficiency but simply aiming at a proper operation of the device (functionality phase). Then, from 2010 to 2019, the trend starts to bend down, taking a definite direction with an improvement of about a factor 10 every 3 years (performance phase); lastly, from 2020 onwards, a significant breaking of the trend-line can be observed, which indicates the difficulty for a further progress of the TDC energy efficiency (saturation phase).

**Figure 19:**
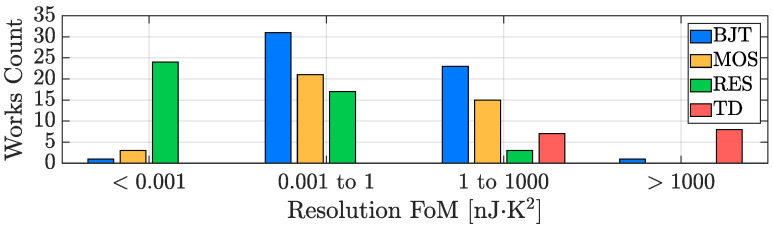
TDC energy efficiency performance distribution with sensing-type distinction.

Similarly to what has been proposed for the previously analyzed TDC parameters of interest, the bar plot of Figure 19 provides an overview of how the different kinds of considered sensing techniques are distributed in terms of energy efficiency. It is clear that, from this point of view, the best performing sensors are the resistor-based ones; BJT-based and MOS-based TDCs offer quite similar performance while, as previously introduced, TD-based TDCs are the most energy-inefficient ones.

### 3.5. Silicon Area

**Figure 20:**
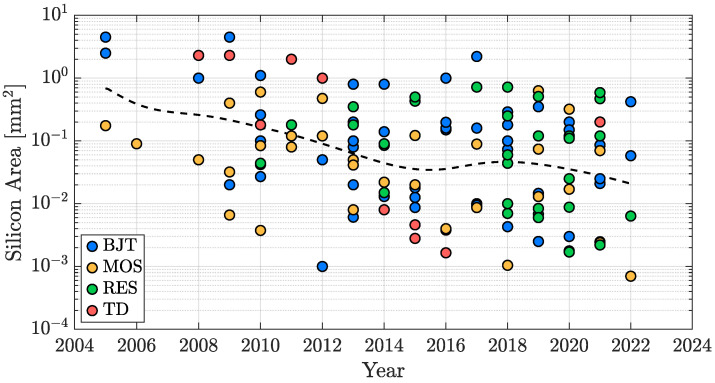
TDC silicon area time evolution in the last two decades.

Finally, the occupied silicon area of the considered TDC works is taken into account; still bearing in mind that it usually offers a direct trade-off with the temperature sensing accuracy performance, the compactness of the TDC is a fundamental requirement considering a production cost minimization perspective. Accordingly, in the last two decades, the size reduction trend has been pretty significant and is shown in Figure 20: it can be observed that the silicon areas of the oldest reported works in the range of 1 mm${}^{2}$ have progressively given way to designs featuring active areas reaching a few hundred of µm${}^{2}$.

**Figure 21:**
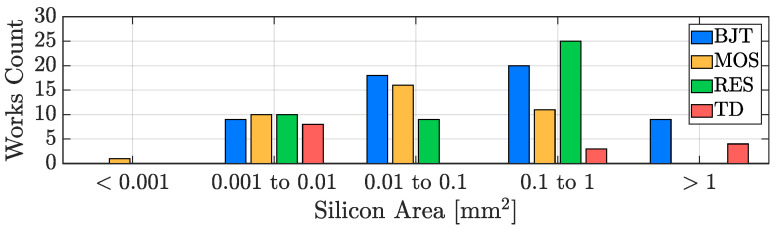
TDC silicon area distribution with sensing-type distinction.

Once more, Figure 21 shows how the considered TDCs are distributed in terms of active area and sensing-type. In this case, as will become clearer in the wrap-up proposed in Section 4, the sensors that, on average, offer the best compactness are the MOS-based ones, followed by the TD-based ones; resistor-based and BJT-based devices, even if there are exceptional cases as [87] or [28], generally require a larger area.

## 4. Conclusions

This paper reviewed the TDCs State-of-the-Art, initially browsing the main on-chip temperature sensing techniques (Section 2) and then highlighting the most significant trends and trade-offs (Section 3).

To summarize the proposed considerations, Table 4 reports performance indicators for each of the four studied sensing techniques and for each of the parameters of interest previously analyzed, with inaccuracy differentiated according to the number of adopted trimming points. For every entry, the geometric mean of the corresponding values of the TDC works discussed in Section 3 has been computed and considered as a meaningful indicator being based on two decades of research activity. For each parameter, the best indicator has been highlighted in green so that the most attractive features of each sensing category could be easily identified; it is interesting to note that, on the basis of a TDC design specifications, each of the sensing techniques could be the optimal choice. Indeed, BJT-based sensors exhibit the best 1-pt trimmed inaccuracy indicator, MOS-based sensors have the lowest conversion energy one and offer the highest degree of compactness, resistor-based sensors feature the best resolution, energy efficiency and accuracy after at least 2 trimming points, while TD-based sensors exhibit the lowest untrimmed inaccuracy.

**Figure 22:**
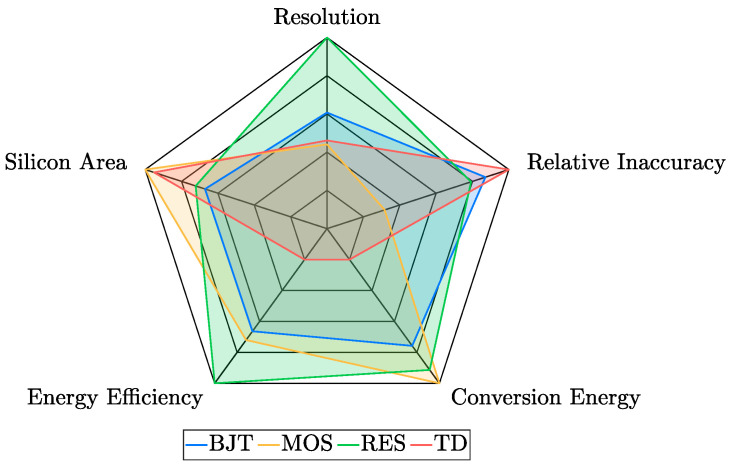
Spider chart summarizing the peculiarities of the four studied categories of TDCs.

Starting from the results collected in Table 4, it has been possible to build a spider chart (Figure 22) to provide a graphical representation of the considerations presented in this work to intuitively and immediately figure out the strengths and the weaknesses of the different categories of TDCs.

To effectively design the spider chart, all the values reported in Table 4 have been normalized with respect to the best one for each parameter of interest (the relative inaccuracy values have been merged according to the coefficients of the rule of thumb introduced in Section 3.2); then, considering that all the parameters are of the lower-is-better kind, they have been converted to a higher-is-better mode with a simple inversion and, finally, have been plotted adopting log-scaled axes to make differences of orders of magnitude still appreciable. Given the extent of the corresponding pentagon, the chart clearly illustrates how promising resistor-based TDCs are and motivates the high number of works exploiting this sensing technique published in the last four years as shown in Section 3. Nevertheless, these kinds of TDCs have considerable linearity issues and, in most cases [28,31,67,68,70,71,72,76,77,78], the employment of nonlinearity polynomial error correction techniques is mandatory; this limit, considering that linearity is a crucial parameter for example in MEMS thermal drift compensation applications, may guide the sensing-type choice to the presented alternatives.

In conclusion, the message is that, since each TDC type excels in a different parameter of interest, the sensing technique should be definitely selected on the basis of the requirements of the specific application for which the TDC is designed for; there is no a priori winner. Finally, the feeling resulting from this review is that the research interest in this field will remain strong in the next several years thanks to a constant need for on-chip temperature sensing in a wide variety of applications and to inherent increasingly challenging requirements. 

## Figures and Tables

**Table 1:** First order TCs of different resistor types in a standard 0.18-µm CMOS process [67].

Resistor Type	TC [K${}^{-1}$]
n+ diffusion	$+1.5\cdot 10^{-3}$
p+ diffusion	$+1.5\cdot 10^{-3}$
n-poly	$-1.5\cdot 10^{-3}$
n-well	$+3.0\cdot 10^{-3}$

**Table 2:** First order TCs of different resistor types in a standard 65-nm CMOS process [68].

Resistor Type	TC [K${}^{-1}$]
n+ diffusion with salicide	$+2.2\cdot 10^{-3}$
n+ diffusion without salicide	$+1.6\cdot 10^{-3}$
n+ poly with salicide	$+2.2\cdot 10^{-3}$
n+ poly without salicide	$+1.2\cdot 10^{-3}$
n-well under oxide diffusion	$+2.5\cdot 10^{-3}$
n-well under shallow trench isolation	$+2.0\cdot 10^{-3}$
p+ diffusion with salicide	$+2.4\cdot 10^{-3}$
p+ diffusion without salicide	$+1.3\cdot 10^{-3}$
p+ poly with salicide	$+2.4\cdot 10^{-3}$
p+ poly without salicide	$-3.2\cdot 10^{-3}$

**Table 3:** Additional details regarding the curves of Figure 6.

α [K${}^{-1}$]	β [K${}^{-1}$]	Sensitivity [mV/K]	Nonlinearity ^1^ [mV]
$+1.5\cdot 10^{-3}$	$-3.0\cdot 10^{-3}$	2.25	126
$+1.5\cdot 10^{-3}$	$-2.25\cdot 10^{-3}$	1.88	52.44
$+1.5\cdot 10^{-3}$	$-1.5\cdot 10^{-3}$	1.5	0
$+1.5\cdot 10^{-3}$	$-0.75\cdot 10^{-3}$	1.13	31.46

^1^ Evaluated as norm of residuals between *V_sig_* and its linear fit across the considered Δ*T* range.

**Table 4:** Performance recap of the different TDC sensing types.

	BJT-Based TDCs	MOS-Based TDCs	Resistor-Based TDCs	TD-Based TDCs
Resolution	48 mK	125 mK	**5 mK**	112 mK
Relative Inaccuracy (untrimmed)	1.95%	/	/	**0.77%**
Relative Inaccuracy (1-pt trimmed)	**0.46%**	2.10 %	1.21%	0.98%
Relative Inaccuracy (at least 2-pt trimmed)	3.47%	1.59%	**0.45%**	/
Conversion Energy	195 nJ	**12 nJ**	51 nJ	121 µJ
Resolution FoM	0.45 nJ·K${}^{2}$	0.18 nJ·K${}^{2}$	**1.43 fJ·K${}^{2}$**	1.53 µJ·K${}^{2}$
Silicon Area	0.085 mm${}^{2}$	**0.033 mm${}^{2}$**	0.073 mm${}^{2}$	0.038 mm${}^{2}$

## Data Availability

Data supporting the reported results can be found at [85].

## Abbreviations

The following abbreviations are used in this manuscript:

ADC	Analog-to-Digital Converter
AFE	Analog Front-End
BJT	Bipolar Junction Transistor
CMOS	Complementary Metal-Oxide-Semiconductor
CTAT	Complementary-To-Absolute-Temperature
DBE	Digital-Back-End
DSP	Digital Signal Processing
DVFS	Dynamic Voltage and Frequency Scaling
ETF	ElectroThermal Filter
FoM	Figure of Merit
IoT	Internet of Things
MEMS	Micro-Electro-Mechanical System
MOSFET	Metal-Oxide-Semiconductor Field-Effect Transistor
OSR	OverSampling Ratio
PTAT	Proportional-To-Absolute-Temperature
REF	REFerence
RES	RESistor
RFID	Radio-Frequency IDentification
SAR	Successive Approximation Register
S/H	Sample and Hold
TC	Temperature Coefficient
TD	Thermal Diffusivity
TDC	Temperature-to-Digital Converter
ZTC	Zero-Temperature-Coefficient
